# High frequency of p53 protein expression in thymic carcinoma but not in thymoma.

**DOI:** 10.1038/bjc.1997.561

**Published:** 1997

**Authors:** N. Hino, K. Kondo, T. Miyoshi, T. Uyama, Y. Monden

**Affiliations:** Second Department of Surgery, School of Medicine, The University of Tokushima, Japan.

## Abstract

**Images:**


British Journal of Cancer (1997) 76(10), 1361–1366
© 1997 Cancer Research Campaign

### High frequency of p53 protein expression in thymic carcinoma but not in thymoma

**N Hino, K Kondo, T Miyoshi, T Uyama and Y Monden**

Second Department of Surgery, School of Medicine, The University of Tokushima, Kuramoto-cho, Tokushima 770, Japan

**Summary** Thymic epithelial tumours are broadly classified into thymomas and thymic carcinomas. Although both tumours occasionally show invasive growth, they exhibit different clinical and biological findings. The oncogene and anti-oncogene in thymic epithelial tumours have not been evaluated fully. We investigated the expression of p53 protein by immunohistochemical analysis using the anti-p53 polyclonal antibody (CM-1) in 17 thymomas and 19 thymic carcinomas. We also examined *p53* gene (exon 5–8) mutation in 18 thymic carcinomas by using polymerase chain reaction–single-strand conformation polymorphism methods and direct sequencing. Of the thymoma cases, only one invasive thymoma showed focal nuclear staining. Fourteen of the 19 thymic carcinomas (74%) showed nuclear staining. Point mutations of the *p53* gene were recognized in only 2 of the 18 thymic carcinomas (11%). One was the mutation C to T transition in the first letter of codon 222 in exon 6, which results in the amino acid substitution from proline to serine. Another was a silent mutation. p53 protein accumulation is highly frequent in thymic carcinomas but not in thymomas, and gene mutation is uncommon in thymic carcinomas.

**Keywords:** thymic carcinoma; thymoma; p53; expression; mutation

Thymic epithelial tumours are broadly classified into thymomas and thymic carcinomas. Thymomas exhibit cytologically bland neoplastic epithelial cells and a variable number of non-neoplastic T-lymphocytes. Thymomas show zonal differentiation, i.e. cortex and medulla elements, which is similar to the normal thymus to some extent (Kodama et al, 1986; Sato et al, 1986; Kondo et al, 1990). Thymomas occasionally show invasive growth and pleural seeding, but lymphogenous or haematogenous metastasis is rare. In contrast, thymic carcinomas do not show zonal differentiation, and their epithelial cells show obvious cytological atypia and are not able to attract and retain immature T-lymphocytes (Kodama et al, 1986; Sato et al, 1986; Kondo et al, 1990). Thymic carcinomas grow invasively and frequently show lymphogenous or haematogenous metastasis. Recent studies have disclosed that thymic carcinomas are different from thymomas in some biological characteristics, i.e. nuclear area, mean nuclear DNA content, DNA histogram pattern and ploidy pattern (Asamura et al, 1988).

In human cancers, accumulation of the p53 protein is probably the most common abnormality (Bartek et al, 1991). Missense mutation of the *p53* gene or some oncoproteins binding to the p53 protein prolongs the half-life of the p53 protein and leads to the accumulation of p53 protein. It also abrogates the ability of normal p53, which suppresses tumour growth (Kuerbitz et al, 1992; Takahashi et al, 1992; Jiang et al, 1993). This may be an important step in the complex process of carcinogenesis in human cancer.

To clarify the relationship between the tumour-suppressor gene *p53* and thymic epithelial tumours, we investigated the expression of p53 protein in thymomas and thymic carcinomas, and the mutation of the *p53* gene in thymic carcinomas.

*Received 7 January 1997*
*Revised 24 April 1997*
*Accepted 1 May 1997*

*Correspondence to:* K Kondo

### MATERIALS AND METHODS

### Materials

Tumour tissues were obtained from 17 thymoma and 19 thymic carcinoma patients who had undergone surgery or biopsy at the Second Department of Surgery, School of Medicine, the University of Tokushima, between 1980 and 1994. All tissues were fixed in formalin and embedded in paraffin wax.

The patients with thymoma included four men and 13 women, whose ages ranged from 29 to 80 years (average 54.8 years). Two patients had myasthenia gravis. The clinical stage of thymoma was determined according to the criteria of Masaoka et al (1981). Eight of the 17 thymomas were non-invasive tumours (clinical stage I). The other nine thymomas were invasive tumours (clinical stage II, III, or IV) (Table 1). The patients with thymic carcinoma included 12 men and seven women, whose ages ranged from 47 to 86 years (average 63.6 years). They had no myasthenia gravis. The histological diagnosis of the tumours was squamous cell carcinoma in 13, spindle cell carcinoma in two, undifferentiated carcinoma in two, adenosquamous cell carcinoma in one and small-cell carcinoma in one (Table 1). The clinical stage of thymic carcinoma was determined according to the criteria of Masaoka et al (1981).

Wild-type *p53* gene from a normal lung tissue was used as a negative control in the polymerase chain reaction–single-strand conformation polymorphism (PCR-SSCP) analysis. Five normal lung and 17 lung carcinoma tissues (seven lung carcinoma tissues with missense mutation in the *p53* gene, one with nonsense mutation and nine with wild-type *p53* gene, which have been reported previously; Kondo et al, 1992) were used as a control in the immunohistochemical staining for p53 protein.

### Immunohistochemical staining for p53

Five-micron-thick paraffin-embedded sections of each thymoma and thymic carcinoma were cut, deparaffinized and rehydrated

**Table 1** Clinical findings in 36 thymic epithelial tumours

| | Thymic carcinoma (*n* = 19) | | Thymoma (*n* = 17) | |
|---|---|---|---|---|
| Age(years) | 63.6 ± 10.97 (47–86) | | 54.8 ± 15.69 (29–80) | |
| Sex | | | | |
| Male | 12 | | 4 | |
| Female | 7 | | 13 | |
| Histology | | | | |
| Sq | 13 | | Lym | 3 |
| Sp | 2 | | Mix | 8 |
| Ud | 2 | | Ep | 6 |
| Sm | 1 | | | |
| Ad | 1 | | | |
| Disease stage | | | | |
| I | 0 | | 8 | |
| II | 2 | | 3 | |
| III | 6 | | 3 | |
| IVa | 2 | | 2 | |
| IVb | 9 | | 1 | |

Sq, squamous cell carcinoma; Sp, spindle cell carcinoma; Ud, undifferentiated carcinoma; Sm, small-cell carcinoma; Ad, adenosquamous cell carcinoma; Lym, lymphocyte-predominant type; Mix, mixed type; Ep, epithelial-predominant type.

through xylene and graded alcohols. For antigen retrieval, the sections were placed in a Coplin jar containing 0.01 M citrate buffer (pH 6.0) and microwaved at 5-min intervals for a total 15 min at maximal level in a household microwave oven (Shi et al, 1991). After heating, the Coplin jar was removed from the microwave oven and allowed to cool. Endogenous peroxidase was inhibited with 3% hydrogen peroxide, and non-specific binding was blocked with bovine serum albumin. Sections were incubated with anti-p53 polyclonal antibody, CM-1 (Novocastra Laboratories, Newcastle, UK) (Midgley et al, 1992) diluted 1:1500 (Fisher et al, 1994) at room temperature for 60 min. After washing with Tris-buffered saline (TBS, pH 7.6), the sections were incubated for 15 min with biotinylated anti-rabbit and anti-mouse immunoglobulins and incubated with streptavidin conjugated to horseradish peroxidase for 15 min using and LSAB kit (Dako, Carpinteria, CA, USA). The peroxidase reaction was developed with a 0.05% solution of diaminobenzidine tetrahydrochloride. Sections were counterstained with haematoxylin. Under light microscopy, we evaluated at least 1000 tumour cells per high-power field. Samples that revealed nuclear staining in more than 10% of the tumour cells were classified as 'positive.' In the evaluation of the p53 protein, we paid no regard to the intensity of the staining because it is dependent on the fixative methods, which may vary among specimens.

### Preparation of DNA

We used paraffin-embedded sections in which the tumour occupied more than 70% of the tissue. Five 10-μm sections were cut and placed in an Eppendorf reaction tube (1.5 ml). These sections were deparaffinized through xylene and graded alcohols. To each of the samples, 400 μl of lysis buffer containing 150 mM sodium chloride, 15 mM sodium citrate, 1% sodium dodecyl sulphate (SDS) and 0.1 mg $ml^{-1}$ of proteinase K was added. The samples were vigorously shaken for 24 h at 48°C. After phenol–chloroform extraction, DNAs were precipitated with cold ethanol for 20 min at –80°C. After centrifugation, the pellets were dried and resuspended in 5–50 μl of distilled water.

### PCR-SSCP analysis

Exons 5–8 of the *p53* gene were investigated by PCR-SSCP methods (Hayashi, 1992). The primer pairs were labelled with [$\gamma$-$^{32}$P]dATP as described previously (Sasa et al, 1993). For amplification, 0.1 μg of tumour DNA was incubated in a total volume of 10 μl of PCR buffer containing 20 mM Tris-HCl, 50 mM potassium chloride, 2 mM magnesium chloride, 200 μM deoxynucleotide triphosphate, 1 μM each of 5′ and 3′ oligonucleotide primers and 0.125 units of DNA Polymerase (Takara Biomedicals, Shiga, Japan). The mixture was overlaid with mineral oil and then amplified. Templates were denatured for 3 min at 95°C, followed by 35 temperature cycles that consisted of 30 s at 94°C, 30 s at 55°C and 60 s at 72°C.

The primer sequences are listed below.

| | |
|---|---|
| Exon 5 | 5′ side: TTCCTCTTCCTGCAGTAC |
| | 3′ side: GCCCCAGCTGCTCACCATCG |
| Exon 6 | 5′ side: CCTCACTGATTGCTCTTAGG |
| | 3′ side: ACCCCAGTTGCAAACCAGAC |
| Exon 7 | 5′ side: CTCCTAGGTTGGCTCTGACT |
| | 3′ side: CAAGTGGCTCCTGACCTGGA |
| Exon 8 | 5′ side: CCTATCCTGAGTAGTGGTAA |
| | 3′ side: GTCCTGCTTGCTTACCTCGC |

The amplified and labelled DNA fragments thus obtained were subjected to electrophoresis at 40 W for 1–4 h in a 6% non-denatured polyacrylamide gel, with or without 10% glycerol, at room temperature. The gel was dried on 3 MM paper (Whatman, Maidstone, UK) and exposed to radiographic film at –80°C for 1 to 12 h, with an intensifying screen.

### Direct sequencing

The extra bands revealed by SSCP analysis were excised. The DNAs were extracted from the extra bands and amplified by PCR. The PCR products were purified using a Mermaid Kit (BIO 101, La Jolla, CA, USA) and directly sequenced using the *Taq*uence Cycle-Sequencing Kit (Biochemical, Cleveland, OH, USA). The PCR products amplified separately from the same sample were also directly sequenced at least twice.

### RESULTS

### Immunohistochemical analysis

*Normal lungs and lung carcinomas*

We analysed p53 expression in the 17 lung carcinomas and five normal lung tissues with and without antigen retrieval by microwave treatment.

Two of the nine lung carcinomas with wild-type *p53* (22%) and four of the seven lung carcinomas with *p53* missense mutation (57%) stained positively in immunohistochemical analysis without microwave treatment. In immunohistochemical analysis with microwave treatment, three of the nine lung carcinomas with wild-type *p53* (33%) and all seven lung carcinomas with *p53* missense mutation (100%) stained positively (Figure 1A).

© Cancer Research Campaign 1997

**Figure 1** The staining patterns in p53 immunohistochemistry with antibody CM-1. (**A**) Lung carcinoma with *p53* mutation shows diffuse nuclear staining. (**B**) Invasive thymoma with atypia shows expression of p53 protein (arrows) on the border of the tumour. (**C**) Nuclear staining of a majority of nuclei is present in thymic carcinoma. (**D**) Nuclear staining is not shown in thymic carcinoma. Bar = 50 μm

The p53 expression detected in the lung carcinomas by immunohistochemical analysis with microwave treatment was significantly correlated with the *p53* gene missense mutation ($P < 0.05$) by Fisher's exact probability test.

The one lung carcinoma with *p53* nonsense mutation and all five normal lungs showed an absence of nuclear staining in the immunohistochemical analysis both with and without microwave treatment.

### *Thymomas*

We analysed the p53 expression in the eight non-invasive thymomas and the nine invasive thymomas. When we did not perform the microwave treatment, no thymomas were stained positively. Even with the microwave treatment, only one of the 17 thymomas was positive for p53 expression. p53 was focally stained on the border of the tumour (Figure 1B). This case was diagnosed as 'invasive thymoma, atypia type combined thymic carcinoma'.

### *Thymic carcinomas*

The numbers of p53-positive cases among the 19 thymic carcinomas with and without microwave treatment were 14 (74%) and 3 (16%) respectively (Figure 1C and D). The positive rate of staining was 100% (two out of two) in stage II, 67% (four out of six) in stage III and 73% (8 out of 11) in stage IV. The expression of p53 protein in the thymic carcinoma tissues was not correlated with the clinical stage. Five cases showed nuclear positive staining in more than 50% of the tumour cells.

### *p53* gene mutation in thymic carcinomas

As it is generally reported that the expression of p53 protein is correlated with missense mutations of the *p53* gene, we examined *p53* gene mutations in the 18 thymic carcinomas by PCR-SSCP and direct sequencing methods. Exons 5–8 of the *p53* gene were amplified by PCR, and the PCR products were analysed by SSCP.

The DNAs of 2 of the 18 thymic carcinomas showed different mobilities from those of the normal lung in the SSCP analysis. Each of these two samples gave four bands: two with the same mobilities as those of the normal lung, corresponding to the two strands of the normal allele, and two other bands with different mobilities, representing the two strands of an aberrant allele (Figure 2A). To confirm the mutation, we performed direct sequencing.

In one case, a sequencing ladder of the variant bands demonstrated the mutation CCG to TCG transition in codon 222 in exon 6, which results in the amino acid substitution from proline to serine (Figure 2B). This sample showed nuclear staining for p53 protein in the immunohistochemical study with microwave treatment. In the other case, a sequencing ladder of the variant bands

© Cancer Research Campaign 1997 

**Figure 2** (**A**) Results of PCR-SSCP analysis for exon 6. N is a wild-type *p53* gene from normal lung; others are from thymic carcinomas. Lane 10 shows bands with different mobility (arrows) from normal bands. (**B**) Direct sequencing of the PCR product from lane 10, which shows a mobility shift, demonstrated the mutation C to T transition in the first letter of codon 222 in exon 6, which results in the amino acid substitution from proline to serine revealed the mutation CAC to CAT transition in codon 178 in exon 5, which was a silent mutation (data not shown); this case did not show nuclear positive staining for p53 protein in the immunohistochemical study with and without microwave treatment.

### DISCUSSION

Thymic carcinoma has long been the source of controversy because of the lack of agreement regarding its definition and proper criteria for diagnosis. Since Shimosato et al (1977) reported eight cases of primary squamous cell carcinoma of the thymus, there have been many reports of thymic carcinoma (Wick et al, 1982; Truong et al, 1990; Suster and Rosai, 1991). Thymic epithelial tumours are broadly classified into thymomas and thymic carcinomas. We define thymic carcinoma as a neoplasm of thymic epithelial cells that exhibits cytological atypia and is not associated with non-neoplastic immature T-lymphocytes, in accord with Shimosato (1994).

It is reported that some fixation methods weaken the antigenicity of the p53 protein (Fisher et al, 1994). In the present study's immunohistochemical analysis without antigen retrieval

**Figure 3** The immunohistochemical expression of p53 protein in thymic epithelial tumours with microwave treatment. *Cases in which more than 50% of tumour cells were stained positively by microwave, p53 protein expression in all samples was rare and weak compared with that in the analysis with antigen retrieval. Although the mechanism of recovering antigenicity by microwave heating is not clear, it is possible that the cross-linking of proteins caused by formaldehyde is altered by microwaves (Shi et al, 1991). We conclude that the results of our immunohistochemical analysis with antigen retrieval by microwave are more reliable than those without antigen retrieval.

We examined the p53 protein expression in the thymic epithelial tumours by immunohistochemical analysis using the polyclonal antibody (CM-1) with antigen retrieval by microwave. Only one of the 17 thymomas (6%) was stained positively by p53 antibody. In contrast, 14 of the 19 thymic carcinomas (74%) were stained positively (Figure 3). Tateyama et al (1995) reported that 57% of the thymomas and 100% of the thymic carcinomas examined showed p53 expression by an anti-p53 antibody, DO-7. None of 21 thymomas and 4 of 13 thymic carcinomas showed nuclear positive staining in more than 50% of the tumour cells. Hayashi et al (1995) demonstrated that the positive rate of p53 immunoreactivity by an anti-p53 antibody (BP53-12) was 42% in non-invasive thymomas, 82.4% in malignant thymomas (category I) and 83.3% in malignant thymomas (category II) according to the classification by Rosai (Suster and Rosai, 1991). Chen et al (1996) reported that three of five (60%) non-invasive thymomas, 8 of 18 (44%) invasive thymomas and 12 of 17 (71%) thymic carcinomas were positive for p53 immunostaining by an anti-p53 antibody, PAb1801. Although the immunoreactivity of thymic carcinoma in the present study was similar to that of their studies, the immunoreactivity of thymoma in the present study was lower than that of their studies. The difference may be due to the sensitivity of the anti-p53 protein antibody used. For example, DO-7 (which Tateyama et al (1995) used) or PAb1801 (which Chen et al (1996) used) is more sensitive than CM-1 for the immunohistochemical analysis of the p53 protein (Friedrichs et al, 1993; Baas et al, 1994). The sensitivity of the anti-p53 protein antibody used may make the difference in p53 expression between thymomas and thymic carcinomas unclear. Gilhus et al (1995) reported that none of the cells in the sections from 24 thymomas were stained for p53

© Cancer Research Campaign 1997

protein with any of the three antibodies DO-7, p240 and PAb1801. Gilhus's result was almost identical to our result. These studies support our finding that the thymic carcinomas showed a higher p53-positive rate than that of the thymomas. The p53 protein accumulation interferes with the ability of wild-type *p53* to inhibit tumour growth (Kuerbitz et al, 1992; Jiang et al, 1993). According to this principle, thymic carcinomas have a tendency to be more proliferative than thymomas.

It is very interesting that the single thymoma case positive for p53 protein in the present study showed characteristics of both thymomas and thymic carcinomas. The tumour showed marked nuclear atypia accompanied by immature T-lymphocytic infiltration (CD1a antibody-positive lymphocytes). Similar cases were reported by Shimosato et al (1994) and Kirchner et al (1992). Shimosato (1994) diagnosed these tumours as 'invasive thymoma, atypical type', and Kirchner diagnosed them as 'well-differentiated thymic carcinoma' (Kirchner et al, 1992).

It has been reported that p53 protein accumulation is caused by missense mutation of the *p53* gene or by interaction of p53 protein with some oncoproteins. In order to clarify the cause of p53 expression in thymic carcinomas, we examined 18 thymic carcinomas for mutation in exons 5–8 of the *p53* gene by PCR-SSCP and direct sequence methods. The missense mutation was found in only one case of the 18 thymic carcinomas. This discrepancy between gene mutation and accumulation of the p53 protein has been reported in some types of lymphomas. In non-HTLV-I associated post-thymic T-cell lymphoma, p53 protein overexpression was detected in 17 of 34 cases, while *p53* mutations were detected in only 3 (17.6%) of these 17 cases (Villuendas et al, 1993). This discrepancy has also been reported in non-Hodgkin's lymphomas and anaplastic large-cell lymphoma (Cesarman et al, 1993; Matsushima et al, 1994). Although there is the possibility that the mutation may be in a portion of the gene not evaluated, mutations of the region other than at exons 5–8 are infrequent in the previous reports. Hollstein et al (1991) demonstrated that the majority of the missense mutations are at codons (exons 5–8) corresponding to amino acids conserved in diverse types of human cancer. We suggest that the expression of p53 protein in thymic carcinomas might be due to interactions with oncoproteins rather than missense mutations, and that a certain oncoprotein might interact with p53 protein in thymic carcinomas and lead to the stabilization of wild-type p53 protein.

Murine double minute 2 (MDM2) protein is known to bind to p53 protein and inhibit p53-mediated transactivation (Momand et al, 1992). We examined the expression of MDM2 using the monoclonal antibody 1B10 (Novocastra Laboratories, Newcastle, UK) (Otto et al, 1993) for MDM2 protein in the thymic carcinoma. In only one of ten thymic carcinomas, the nucleus was stained diffusely (data not shown). The MDM2 protein expression in the thymic carcinomas seems not to relate to p53 protein expression.

Takeyama et al (1995) reported that all tumours (eight thymomas and two thymic carcinomas) that they examined had missense mutations in the *p53* gene, and that three of these tumours were focally stained by anti-p53 antibody (a positive rate of <10%). This is a surprisingly high rate compared with other human cancers. Weirich et al (1996) reported two (13%) missense mutations and two (13%) silent mutation cases among 16 thymic carcinomas and no mutations in 28 thymomas detected by PCR-SSCP analysis and sequencing methods. Weirich's results are similar to ours. The sensitivity of PCR-SSCP for detecting point mutations is more than 89% for 300- to 400-bp fragments, and the specificity is 100% (Hayashi, 1992). We believe that *p53* gene mutations in thymic epithelial tumours are rare.

The overexpression of the p53 protein in lung cancer in the present study was almost as frequent as that in the thymic carcinomas. Most of the lung cancers with p53 protein expression had missense mutations. In contrast, few thymic carcinomas with p53 protein expression had missense mutations. p53 protein expression without missense mutation might be one of the differential diagnostic factors between thymic carcinoma and lung cancer.

In conclusion, p53 protein accumulation was highly frequent in the thymic carcinomas but not in the thymomas, while gene mutation was uncommon in the thymic carcinomas. We suggest that the accumulation of p53 protein may correlate with the difference in the malignant potential between thymic carcinomas and thymomas. This characteristic may be one of the differential diagnostic factors between thymic carcinoma and lung cancer.

### REFERENCES

Asamura H, Nakajima T, Mukai K, Noguchi M and Shimosato Y (1988) Degree of malignancy of thymic epithelial tumors in terms of nuclear DNA content and nuclear area. An analysis of 39 cases. *Am J Pathol* **133**: 615–622

Baas IO, Mulder JW, Offerhaus GJ, Vogelstein B and Hamilton SR (1994) An evaluation of six antibodies for immunohistochemistry of mutant p53 gene product in archival colorectal neoplasms. *J Pathol* **172**: 5–12

Bartek J, Bartkova J, Vojtesek B, Staskova Z, Lukas J, Rejthar A, Kovarik J, Midgley CA, Gannon JV and Lane DP (1991) Aberrant expression of the p53 oncoprotein is a common feature of a wide spectrum of human malignancies. *Oncogene* **6**: 1699–1703

Cesarman E, Inghirami G, Chadburn A and Knowles DM (1993) High levels of p53 protein expression do not correlate with p53 gene mutations in anaplastic large cell lymphoma. *Am J Pathol* **143**: 845–856

Chen FF, Yan JJ, Jin YT and Su IJ (1996) Detection of bcl-2 and p53 in thymoma: expression of bcl-2 as a reliable marker of tumor aggressiveness. *Hum Pathol* **27**: 1089–1092

Fisher CJ, Gillett CE, Vojtesek B, Barnes DM and Millis RR (1994) Problems with p53 immunohistochemical staining: the effect of fixation and variation in the methods of evaluation. *Br J Cancer* **69**: 26–31

Friedrichs K, Gliba S, Eidtmann H and Jonat W (1993) Overexpression of p53 and prognosis in breast cancer. *Cancer* **72**: 3641–3647

Gilhus NE, Jones M, Turley H, Gatter KC, Nagvekar N, Newsom DJ and Willcox N (1995) Oncogene proteins and proliferation antigens in thymomas: increased expression of epidermal growth factor receptor and Ki67 antigen. *J Clin Pathol* **48**: 447–455

Hayashi K (1992) PCR-SSCP: a method for detection of mutations. *Genet Anal Tech Appl* **9**: 73–79

Hayashi Y, Ishii N, Obayashi C, Jinnai K, Hahioka K, Imai Y and Itoh H (1995) Thymoma: tumour type related to expression of epidermal growth factor (EGF), EGF-receptor, p53, v-erb B and ras p21. *Virchows Arch* **426**: 43–50

Hollstein MD, Sidransky B, Vogelstein B and Harris CC (1991) p53 mutations in human cancers. *Science* **253**: 49–53

Jiang D, Srinivasan A, Lozano G and Robbins PD (1993) SV40 T antigen abrogates p53-mediated transcriptional activity. *Oncogene* **8**: 2805–2812

Kirchner T, Schalke B, Buchwald J, Ritter M, Marx A and Müller HH (1992) Well-differentiated thymic carcinoma. An organotypical low-grade carcinoma with relationship to cortical thymoma. *Am J Surg Pathol* **16**: 1153–1169

Kodama T, Watanabe S, Sato Y, Shimosato Y and Miyazawa N (1986) An immunohistochemical study of thymic epithelial tumors. I. Epithelial component. *Am J Surg Pathol* **10**: 26–33

Kondo K, Mukai K, Sato Y, Matsuno Y, Shimosato Y and Monden Y (1990) An immunohistochemical study of thymic epithelial tumors. III. The distribution of interdigitating reticulum cells and S-100b-positive small lymphocytes. *Am J Surg Pathol* **14**: 1139–1147

Kondo K, Umemoto A, Akimoto S, Uyama T, Hayashi K, Ohnishi Y and Monden Y (1992) Mutations in the P53 tumour suppressor gene in primary lung cancer in Japan. *Biochem Biophys Res Commun* **183**: 1139–1146

Kuerbitz SJ, Plunkett BS, Walsh WV and Kastan MB (1992) Wild-type p53 is a cell cycle checkpoint determinant following irradiation. *Proc Natl Acad Sci USA* **89**: 7491–7495

© Cancer Research Campaign 1997

Masaoka A, Monden Y, Nakahara K and Tanioka T (1981) Follow-up study of thymomas with special reference to their clinical stages. *Cancer* **48**: 2485–2492

Matsushima AY, Cesarman E, Chadburn A and Knowles DM (1994) Post-thymic T cell lymphomas frequently overexpress p53 protein but infrequently exhibit p53 gene mutations. *Am J Pathol* **144**: 573–584

Midgley CA, Fisher CJ, Bartek J, Vojtesek B, Lane D and Barnes DM (1992) Analysis of p53 expression in human tumours: an antibody raised against human p53 expressed in *Escherichia coli*. J Cell Sci **101**: 183–189

Momand J, Zambetti GP, Olson DC, George D and Levine AJ (1992) The mdm-2 oncogene product forms a complex with the p53 protein and inhibits p53-mediated transactivation. *Cell* **69**: 1237–1245

Otto A and Deppert W (1993) Upregulation of mdm-2 expression in Meth A tumor cells tolerating wild-type p53. *Oncogene* **8**: 2591–2603

Sasa M, Kondo K, Komaki K, Uyama T, Morimoto T and Monden Y (1993) Frequency of spontaneous p53 mutations (CpG site) in breast cancer in Japan. *Breast Cancer Res Treat* **27**: 247–252

Sato Y, Watanabe S, Mukai K, Kodama T, Upton MP, Goto M and Shimosato Y (1986) An immunohistochemical study of thymic epithelial tumors. II. Lymphoid component. *Am J Surg Pathol* **10**: 862–870

Shi SR, Key ME and Kalra KL (1991) Antigen retrieval in formalin-fixed, paraffin-embedded tissues: an enhancement method for immunohistochemical staining based on microwave oven heating of tissue sections. *J Histochem Cytochem* **39**: 741–748

Shimosato Y (1994) Controversies surrounding the subclassification of thymoma. *Cancer* **74**: 542–544

Shimosato Y, Kameya T, Nagai K and Suemasu K (1977) Squamous cell carcinoma of the thymus. An analysis of eight cases. *Am J Surg Pathol* **1**: 109–121

Suster S and Rosai J (1991) Thymic carcinoma. A clinicopathologic study of 60 cases. *Cancer* **67**: 1025–1032

Takahashi T, Carbone D, Takahashi T, Nau MM, Hida T, Linnoila I, Ueda R and Minna JD (1992) Wild-type but not mutant p53 suppresses the growth of human lung cancer cells bearing multiple genetic lesions. *Cancer Res* **52**: 2340–2343

Tateyama H, Eimoto T, Tada T, Mizuno T, Inagaki H, Hata A, Sasaki M and Masaoka A (1995) p53 protein expression and p53 gene mutation in thymic epithelial tumors. An immunohistochemical and DNA sequencing study. *Am J Clin Pathol* **104**: 375–381

Truong LD, Mody DR, Cagle PT, Jackson YG, Schwartz MR and Wheeler TM. (1990). Thymic carcinoma. A clinicopathologic study of 13 cases. *Am J Surg Pathol* **14**: 151–166

Villuendas R, Piris MA, Algara P, Sanchez BM, Sanchez VL, Martinez JC, Orradre JL, Garcia P, Lopez C and Martinez P (1993) The expression of p53 protein in non-Hodgkin's lymphomas is not always dependent on p53 gene mutations. *Blood* **82**: 3151–3156

Weirich G, Schneider P, Fellbaum C, Nathrath W, Brauch H, Präuer H and Höfler H (1996) P53-alterations in thymic epithelial tumours (abstracts). *First Conference on Biological and Clinical Aspects of Thymic Epithelial Tumors, Würzburg*. Müler-Hesmelink (ed.). Pathology Institute of University of Würzburg: Würzburg

 *© Cancer Research Campaign 1997*


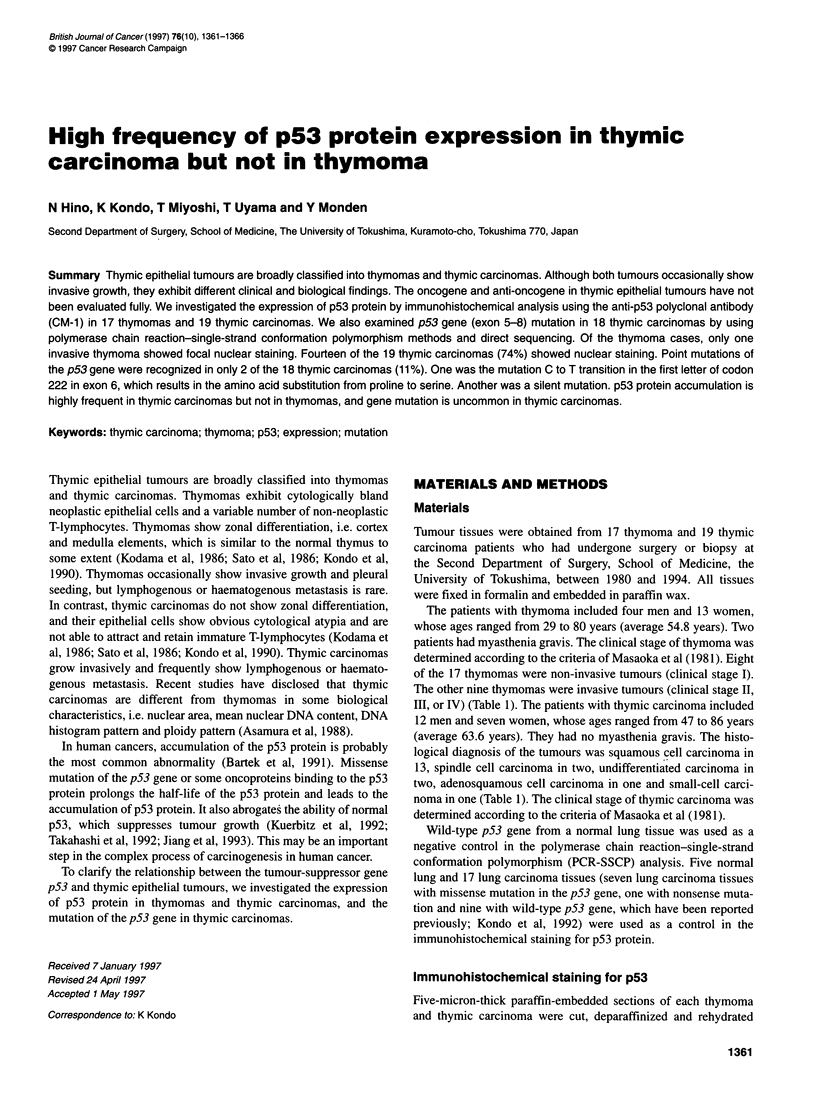

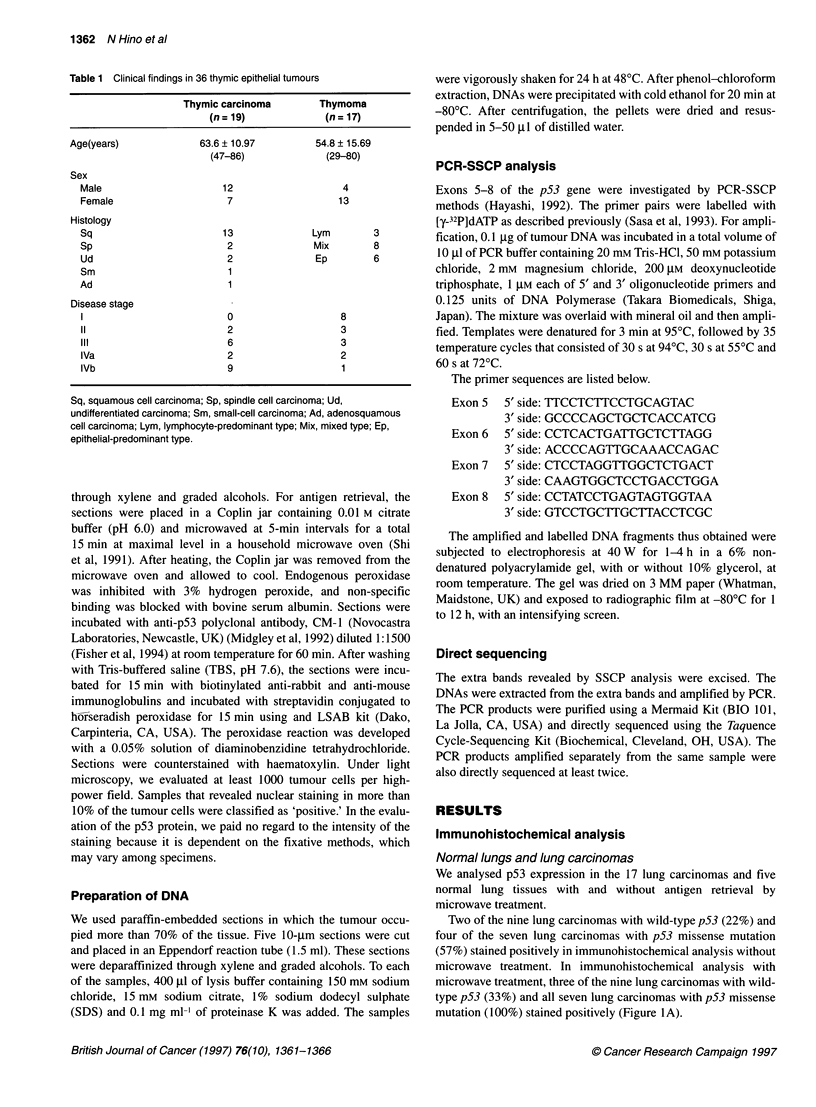

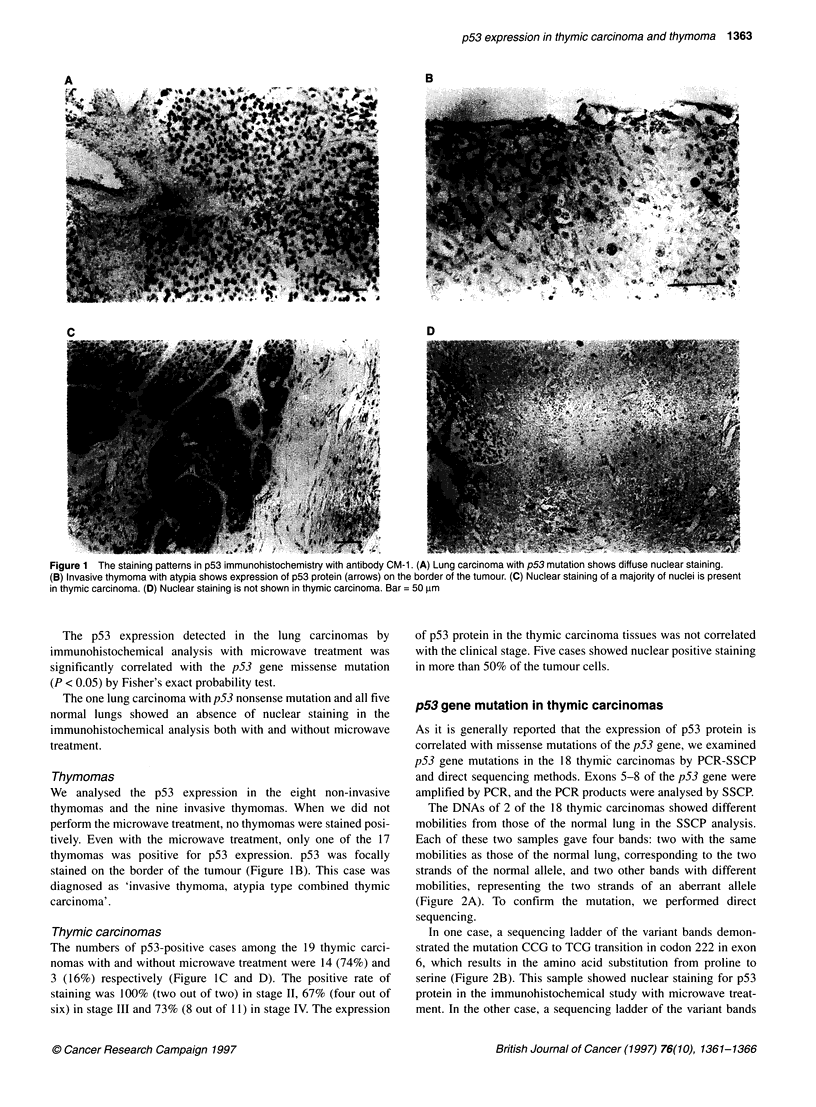

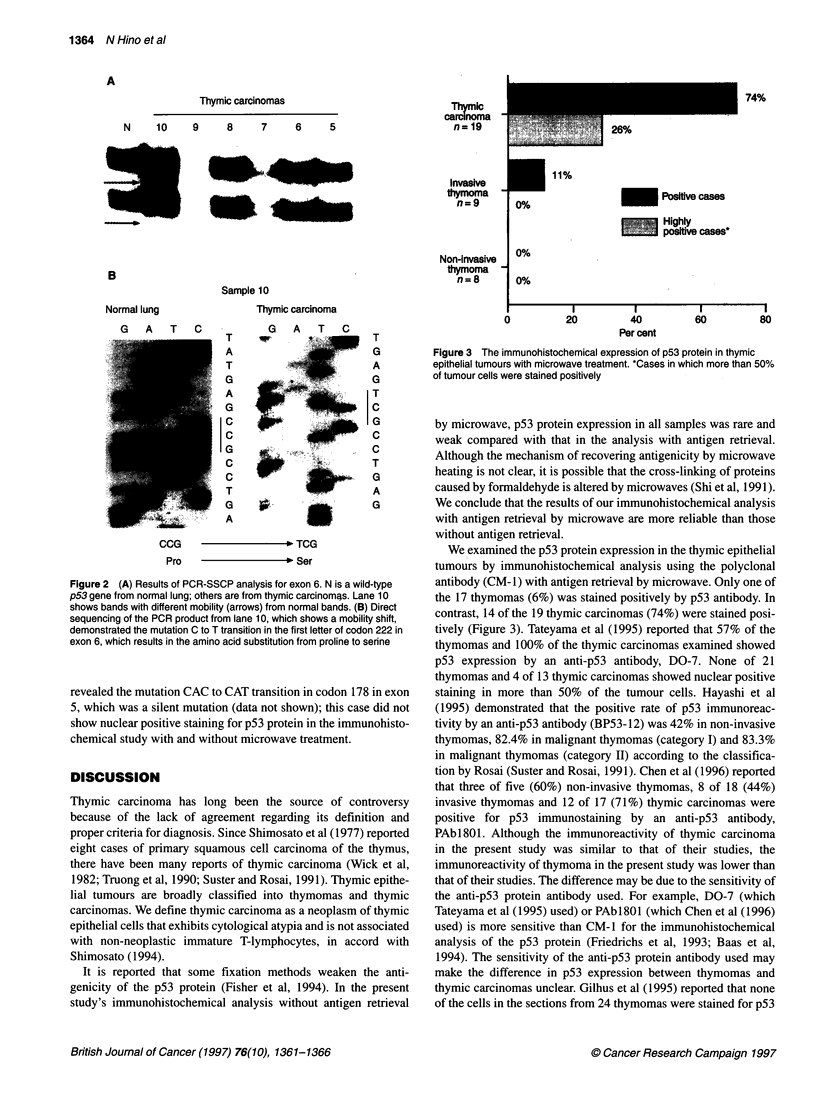

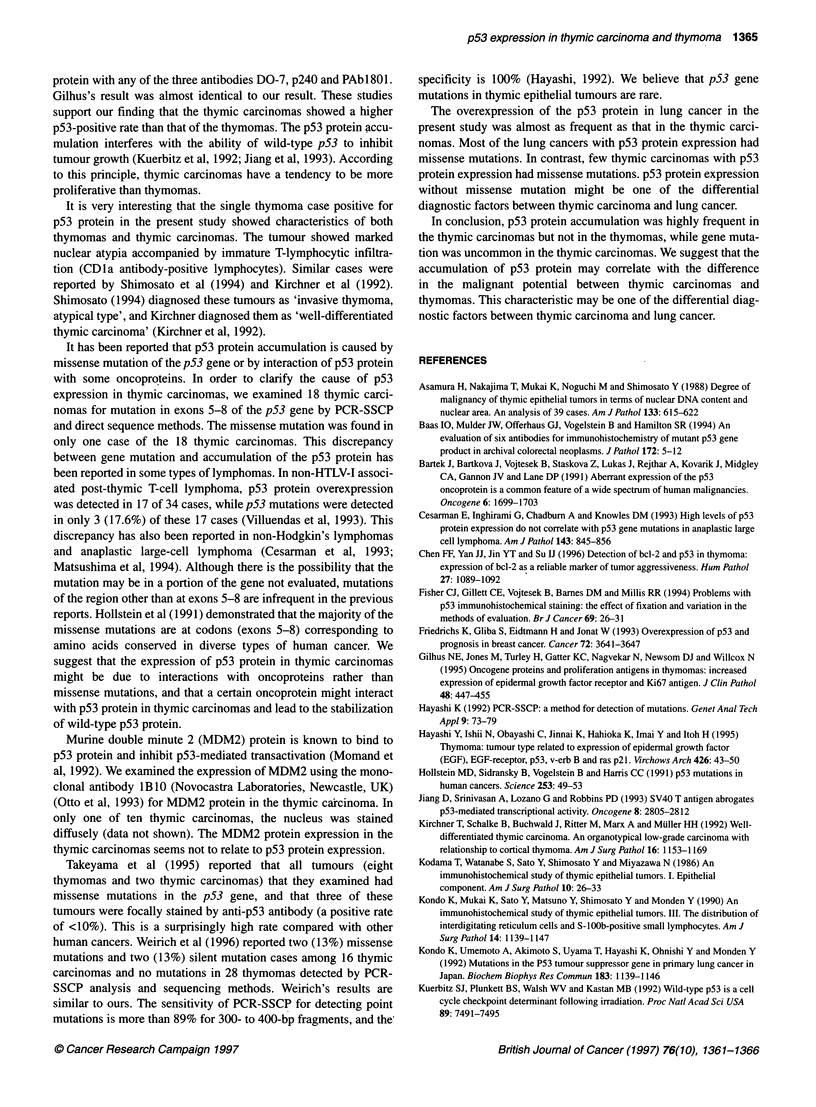

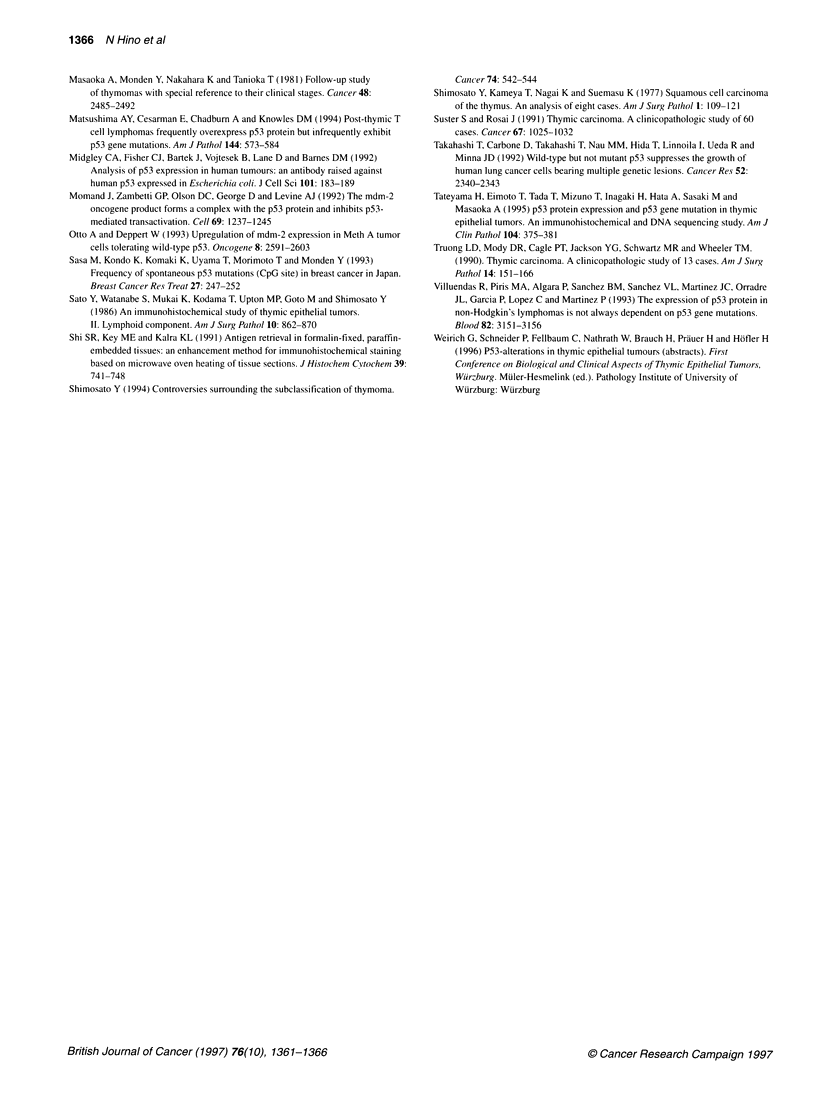


## References

[OCR_00577] Asamura H., Nakajima T., Mukai K., Noguchi M., Shimosato Y. (1988). Degree of malignancy of thymic epithelial tumors in terms of nuclear DNA content and nuclear area. An analysis of 39 cases.. Am J Pathol.

[OCR_00582] Baas I. O., Mulder J. W., Offerhaus G. J., Vogelstein B., Hamilton S. R. (1994). An evaluation of six antibodies for immunohistochemistry of mutant p53 gene product in archival colorectal neoplasms.. J Pathol.

[OCR_00587] Bártek J., Bártková J., Vojtesek B., Stasková Z., Lukás J., Rejthar A., Kovarík J., Midgley C. A., Gannon J. V., Lane D. P. (1991). Aberrant expression of the p53 oncoprotein is a common feature of a wide spectrum of human malignancies.. Oncogene.

[OCR_00594] Cesarman E., Inghirami G., Chadburn A., Knowles D. M. (1993). High levels of p53 protein expression do not correlate with p53 gene mutations in anaplastic large cell lymphoma.. Am J Pathol.

[OCR_00599] Chen F. F., Yan J. J., Jin Y. T., Su I. J. (1996). Detection of bcl-2 and p53 in thymoma: expression of bcl-2 as a reliable marker of tumor aggressiveness.. Hum Pathol.

[OCR_00604] Fisher C. J., Gillett C. E., Vojtesek B., Barnes D. M., Millis R. R. (1994). Problems with p53 immunohistochemical staining: the effect of fixation and variation in the methods of evaluation.. Br J Cancer.

[OCR_00609] Friedrichs K., Gluba S., Eidtmann H., Jonat W. (1993). Overexpression of p53 and prognosis in breast cancer.. Cancer.

[OCR_00613] Gilhus N. E., Jones M., Turley H., Gatter K. C., Nagvekar N., Newsom-Davis J., Willcox N. (1995). Oncogene proteins and proliferation antigens in thymomas: increased expression of epidermal growth factor receptor and Ki67 antigen.. J Clin Pathol.

[OCR_00620] Hayashi K. (1992). PCR-SSCP: a method for detection of mutations.. Genet Anal Tech Appl.

[OCR_00624] Hayashi Y., Ishii N., Obayashi C., Jinnai K., Hanioka K., Imai Y., Itoh H. (1995). Thymoma: tumour type related to expression of epidermal growth factor (EGF), EGF-receptor, p53, v-erb B and ras p21.. Virchows Arch.

[OCR_00629] Hollstein M., Sidransky D., Vogelstein B., Harris C. C. (1991). p53 mutations in human cancers.. Science.

[OCR_00633] Jiang D., Srinivasan A., Lozano G., Robbins P. D. (1993). SV40 T antigen abrogates p53-mediated transcriptional activity.. Oncogene.

[OCR_00637] Kirchner T., Schalke B., Buchwald J., Ritter M., Marx A., Müller-Hermelink H. K. (1992). Well-differentiated thymic carcinoma. An organotypical low-grade carcinoma with relationship to cortical thymoma.. Am J Surg Pathol.

[OCR_00642] Kodama T., Watanabe S., Sato Y., Shimosato Y., Miyazawa N. (1986). An immunohistochemical study of thymic epithelial tumors. I. Epithelial component.. Am J Surg Pathol.

[OCR_00647] Kondo K., Mukai K., Sato Y., Matsuno Y., Shimosato Y., Monden Y. (1990). An immunohistochemical study of thymic epithelial tumors. III. The distribution of interdigitating reticulum cells and S-100 beta-positive small lymphocytes.. Am J Surg Pathol.

[OCR_00653] Kondo K., Umemoto A., Akimoto S., Uyama T., Hayashi K., Ohnishi Y., Monden Y. (1992). Mutations in the P53 tumour suppressor gene in primary lung cancer in Japan.. Biochem Biophys Res Commun.

[OCR_00658] Kuerbitz S. J., Plunkett B. S., Walsh W. V., Kastan M. B. (1992). Wild-type p53 is a cell cycle checkpoint determinant following irradiation.. Proc Natl Acad Sci U S A.

[OCR_00667] Masaoka A., Monden Y., Nakahara K., Tanioka T. (1981). Follow-up study of thymomas with special reference to their clinical stages.. Cancer.

[OCR_00672] Matsushima A. Y., Cesarman E., Chadburn A., Knowles D. M. (1994). Post-thymic T cell lymphomas frequently overexpress p53 protein but infrequently exhibit p53 gene mutations.. Am J Pathol.

[OCR_00677] Midgley C. A., Fisher C. J., Bártek J., Vojtesek B., Lane D., Barnes D. M. (1992). Analysis of p53 expression in human tumours: an antibody raised against human p53 expressed in Escherichia coli.. J Cell Sci.

[OCR_00682] Momand J., Zambetti G. P., Olson D. C., George D., Levine A. J. (1992). The mdm-2 oncogene product forms a complex with the p53 protein and inhibits p53-mediated transactivation.. Cell.

[OCR_00687] Otto A., Deppert W. (1993). Upregulation of mdm-2 expression in Meth A tumor cells tolerating wild-type p53.. Oncogene.

[OCR_00691] Sasa M., Kondo K., Komaki K., Uyama T., Morimoto T., Monden Y. (1993). Frequency of spontaneous p53 mutations (CpG site) in breast cancer in Japan.. Breast Cancer Res Treat.

[OCR_00696] Sato Y., Watanabe S., Mukai K., Kodama T., Upton M. P., Goto M., Shimosato Y. (1986). An immunohistochemical study of thymic epithelial tumors. II. Lymphoid component.. Am J Surg Pathol.

[OCR_00701] Shi S. R., Key M. E., Kalra K. L. (1991). Antigen retrieval in formalin-fixed, paraffin-embedded tissues: an enhancement method for immunohistochemical staining based on microwave oven heating of tissue sections.. J Histochem Cytochem.

[OCR_00708] Shimosato Y. (1994). Controversies surrounding the subclassification of thymoma.. Cancer.

[OCR_00712] Shimosato Y., Kameya T., Nagai K., Suemasu K. (1977). Squamous cell carcinoma of the thymus. An analysis of eight cases.. Am J Surg Pathol.

[OCR_00716] Suster S., Rosai J. (1991). Thymic carcinoma. A clinicopathologic study of 60 cases.. Cancer.

[OCR_00720] Takahashi T., Carbone D., Takahashi T., Nau M. M., Hida T., Linnoila I., Ueda R., Minna J. D. (1992). Wild-type but not mutant p53 suppresses the growth of human lung cancer cells bearing multiple genetic lesions.. Cancer Res.

[OCR_00726] Tateyama H., Eimoto T., Tada T., Mizuno T., Inagaki H., Hata A., Sasaki M., Masaoka A. (1995). p53 protein expression and p53 gene mutation in thymic epithelial tumors. An immunohistochemical and DNA sequencing study.. Am J Clin Pathol.

[OCR_00733] Truong L. D., Mody D. R., Cagle P. T., Jackson-York G. L., Schwartz M. R., Wheeler T. M. (1990). Thymic carcinoma. A clinicopathologic study of 13 cases.. Am J Surg Pathol.

[OCR_00738] Villuendas R., Piris M. A., Algara P., Sánchez-Beato M., Sánchez-Verde L., Martinez J. C., Orradre J. L., García P., Lopez C., Martinez P. (1993). The expression of p53 protein in non-Hodgkin's lymphomas is not always dependent on p53 gene mutations.. Blood.

